# Delineating memory reactivation in sleep with verbal and non-verbal retrieval cues

**DOI:** 10.1093/cercor/bhae183

**Published:** 2024-05-14

**Authors:** Anna á V Guttesen, Dan Denis, M Gareth Gaskell, Scott A Cairney

**Affiliations:** Department of Psychology, University of York, York YO10 5DD, United Kingdom; Wellcome Centre for Integrative Neuroimaging, Nuffield Department of Clinical Neurosciences, University of Oxford, Oxford OX3 9DU, United Kingdom; Department of Psychology, University of York, York YO10 5DD, United Kingdom; York Biomedical Research Institute, University of York, York YO10 5DD, United Kingdom; Department of Psychology, University of York, York YO10 5DD, United Kingdom; York Biomedical Research Institute, University of York, York YO10 5DD, United Kingdom; Department of Psychology, University of York, York YO10 5DD, United Kingdom; York Biomedical Research Institute, University of York, York YO10 5DD, United Kingdom

**Keywords:** sleep, targeted memory reactivation, auditory retrieval cues, memory consolidation, spindles

## Abstract

Sleep supports memory consolidation via the reactivation of newly formed memory traces. One way to investigate memory reactivation in sleep is by exposing the sleeping brain to auditory retrieval cues; a paradigm known as targeted memory reactivation. To what extent the acoustic properties of memory cues influence the effectiveness of targeted memory reactivation, however, has received limited attention. We addressed this question by exploring how verbal and non-verbal memory cues affect oscillatory activity linked to memory reactivation in sleep. Fifty-one healthy male adults learned to associate visual stimuli with spoken words (verbal cues) and environmental sounds (non-verbal cues). Subsets of the verbal and non-verbal memory cues were then replayed during sleep. The voice of the verbal cues was either matched or mismatched to learning. Memory cues (relative to unheard control cues) prompted an increase in theta/alpha and spindle power, which have been heavily implicated in sleep-associated memory processing. Moreover, verbal memory cues were associated with a stronger increase in spindle power than non-verbal memory cues. There were no significant differences between the matched and mismatched verbal cues. Our findings suggest that verbal memory cues may be most effective for triggering memory reactivation in sleep, as indicated by an amplified spindle response.

## Introduction

Sleep facilitates memory consolidation; the process through which information is retained in long-term memory. Sleep-associated memory gains were initially thought to arise from a passive protective mechanism, whereby sleep shields newly acquired memories from the interference posed by wakefulness (Jenkins and Dallenbach 1924). However, more recent work has suggested that sleep also plays an active role in offline memory processing (Born and Wilhelm 2012; Rasch and Born 2013; Klinzing et al. 2019; Denis and Cairney 2023, 2024), such that hippocampus-dependent memories are repeatedly reactivated and gradually integrated with pre-existing representations in neocortex.

According to this *Active Systems Consolidation* framework, memory processing in sleep relies on finely-tuned interactions between the cardinal neural oscillations of slow-wave sleep (SWS): < 1 Hz neocortical slow oscillations (SOs), 11–16 Hz thalamocortical sleep spindles, and ~ 80–100 Hz hippocampal ripples. Embedded within SOs, sleep spindles are thought to cluster reactivated memory units in the form of ripples to coordinate their transfer from hippocampus to neocortex for long-term storage (Born and Wilhelm 2012; Rasch and Born 2013; Klinzing et al. 2019; Staresina 2024). Several studies in humans have provided compelling support for this view, demonstrating that patterns of brain activity observed at learning re-emerge during spindles, highlighting spindles as a candidate neural marker of memory reactivation in sleep (Bergmann et al. 2012; Schönauer et al. 2017; Cairney et al. 2018a; Wang et al. 2019; Schreiner et al. 2021).

Alongside spindles, SOs, and ripples, the 4–8 Hz theta rhythm has also been implicated in overnight memory consolidation (Schreiner et al. 2018). Adaptations of the Active Systems framework suggest that spindle and theta oscillations work in unison to support memory reactivation and stabilization during SWS, with theta activity representing the initial reactivation of newly formed memories and spindles signifying their subsequent reprocessing and migration to neocortex (Schreiner and Rasch 2017; Antony et al. 2019).

Our understanding of sleep’s role in offline memory processing has been heavily influenced by the development of a memory cueing paradigm known as targeted memory reactivation (TMR; Rudoy et al. 2009). In a typical TMR study, participants form new memories that are associated with sounds at the time of learning. A subset of these sounds is then replayed during SWS to trigger the reactivation of their associated memory traces. A wide range of studies have shown that retention over sleep is improved for memories that are cued by TMR relative to those that are not (Schönauer et al. 2014; Schreiner et al. 2015a; Schreiner and Rasch 2015; Cairney et al. 2016; Antony et al. 2018; Göldi et al. 2019; Hu et al. 2020; Schechtman et al. 2021), providing causal evidence that memory reactivation is a central mechanism of sleep-associated consolidation.

Beyond behavioral manifestations of memory reactivation, TMR has provided important evidence for the role of sleep spindles and theta oscillations in overnight consolidation. In humans, memory cues delivered during SWS trigger a transient increase in spindle activity, with the magnitude of this increase predicting later memory performance (Cox et al. 2014; Schreiner et al*.* 2015a; Lehmann et al. 2016; Farthouat et al. 2017; Groch et al. 2017; Antony et al. 2018; Laventure et al. 2018; Wang et al. 2019; Schechtman et al. 2021). TMR-evoked increases in spindle activity are often preceded by a surge in theta activity, consistent with the view that theta and spindle rhythms play complementary roles in offline memory processing (Schreiner et al*.* 2015a; Schreiner and Rasch 2015; Lehmann et al. 2016; Farthouat et al. 2017; Groch et al. 2017; Oyarzún et al. 2017; Laventure et al. 2018; Bar et al. 2020; Schechtman et al. 2021; Joensen et al. 2022).

Although the majority of TMR studies have used environmental sounds (e.g. a dog barking) as memory cues (Rudoy et al. 2009; Van Dongen et al. 2012; Schönauer et al. 2014; Cairney et al. 2016; Antony et al. 2018; Vargas et al. 2019; Schechtman et al. 2021), several others have delivered verbal stimuli (i.e. spoken words) during sleep (Schreiner et al. 2015b; Schreiner and Rasch 2015; Lehmann et al. 2016; Farthouat et al. 2017; Cairney et al*.* 2018a; Göldi et al. 2019; Joensen et al. 2022). Whether the effectiveness of TMR is any greater for verbal or non-verbal cues has received only limited attention, but is nevertheless an important question: by determining which type of memory cue engenders the greatest impact on offline memory processing, we can optimize TMR protocols and strengthen their potential utility as tools in education and healthcare.

In previous work, we compared the impacts of verbal and non-verbal memory cues on sleep-associated consolidation. Participants associated visual stimuli with spoken words (verbal cues) or environmental sounds (non-verbal cues) at learning, which were then replayed during SWS (Cairney et al. 2017). Although cueing in SWS improved memory retention, the magnitude of this behavioral improvement was highly comparable across the verbal and non-verbal cueing conditions, suggesting that cue type had no impact on the memory enhancing effects of TMR.

However, the coarse behavioral measures used in our prior study (paired associates forgetting) might have been inadequate to detect differences in the effectiveness of verbal and non-verbal TMR cues. Given the recent evidence that spindle activity in sleep provides a neural marker of offline memory replay, sleep spindles (and other neural oscillations linked to memory reactivation in SWS) might provide a more reliable means of indexing the sleeping brain’s responsiveness to different types of memory cue.

Our earlier work also compared the memory effects of verbal TMR when the cues were acoustically matched or mismatched to those encountered at learning. Whereas matched verbal cueing led to a selective improvement in retention for the cued (but not the non-cued) associations, mismatched verbal cueing prompted a performance gain for both the cued and non-cued memories. This suggests that mismatched cueing might promote a generalized form of reactivation across a single learning context, and is in keeping with recent evidence that multiple memories can be simultaneously reactivated in response to a single TMR cue (Schechtman et al. 2022). However, to what extent the varied influences of matched and mismatched TMR cues are driven by disparate neural mechanisms cannot be inferred from our behavioral findings. The brain rhythms implicated in TMR might thus offer a solution to this outstanding question.

We report on a secondary analysis of sleep EEG data acquired in our previous study (Cairney et al. 2017) comparing the effects of TMR with verbal versus non-verbal memory cues (Experiment 1), and acoustically matched versus mismatched verbal memory cues (Experiment 2). Because our TMR protocol included verbal and non-verbal control stimuli that were not presented at learning, we could isolate the time-frequency responses associated with memory reactivation in sleep (i.e. memory cues > control cues; Cairney et al*.* 2018a). We exploited these time-frequency representations in our well-powered sample (*n* = 51) to investigate whether the sleeping brain is more responsive to verbal or non-verbal memory cues (i.e. based on the evoked spindle/theta response), and assess whether the divergent behavioral influences of matched and mismatched cueing observed in our prior work are accompanied by distinct neural activity profiles.

## Materials and methods

### Participants

Data from 51 healthy males were analyzed (Experiment 1: *n* = 28, mean ± SD age = 20.32 ± 1.54, Experiment 2: *n* = 23, mean ± SD age = 20.96 ± 2.38). Screening questionnaires indicated that participants had no history of sleep, neurological or psychiatric disorders, were non-smokers and were not using any psychoactive medications. Only male participants were included due to an ethical committee requirement of at least one experimenter being the same gender as the participants staying in the lab overnight. Because the lead experimenter was male and working alone, only male participants could be included in the sample. Following standard practices in our lab, participants were instructed to refrain from alcohol and caffeine for 24 h before the start of the study (Cairney et al. 2018b; Ashton et al. 2019; Strachan et al. 2020; Ashton and Cairney 2021; Guttesen et al. 2022; Petzka et al. 2023). The Pittsburgh Sleep Quality Index (Buysse et al. 1989) indicated that all participants had a normal pattern of sleep in the month preceding the study. Participants provided written and informed consent and the study was approved by the Research Ethics Committee of the Department of Psychology at the University of York.

### Procedure

#### Experiment 1

##### Evening

**Fig. 1:**
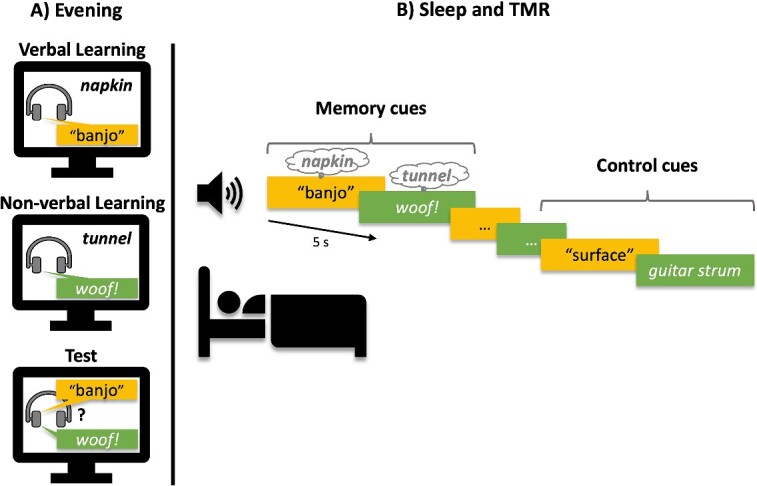
Experimental procedure. (A) Participants learned to associate visually presented words with verbal and non-verbal auditory cues. (B) Verbal and non-verbal auditory cues were replayed during slow-wave sleep (order randomized). Previously unheard control cues were also played. Verbal cues were presented in the same voice as at learning (matched) in Experiment 1 and in a different voice to learning in Experiment 2 (mismatched). Non-verbal TMR cues were identical to those heard at learning in both experiments.

Figure 1 illustrates the experimental procedure. Participants arrived at the sleep laboratory at 9:30 pm (± 30 min) and were wired up for sleep EEG monitoring. They were informed that the study was about the role of sleep in memory consolidation but were not told about the TMR manipulation. Training comprised two paired-associates tasks, one with verbal cues and the other with non-verbal cues (performed separately and order counterbalanced across participants). Both tasks included a learning phase and a test phase. At learning, participants associated each of 28 visually presented words with an auditory stimulus (verbal or non-verbal, depending on the task that they were performing). All verbal cues were presented in a male or female voice (counterbalanced across participants). At test, participants were presented with each of the auditory stimuli again (verbal or non-verbal) and instructed to type the associated words. If participants failed to correctly recall > 60% of the target words, the training (learning and test) was repeated (the mean ± SEM number of training rounds needed to reach criterion was 1.32 ± 0.10 for verbal cue training and 1.79 ± 0.11 for non-verbal cue training; *t*(27) = 3.86; *P* = 0.001). Participants then completed a final pre-sleep test where they were assessed on all 56 verbal and non-verbal paired associates presented in random order (following the same procedures as the prior test phases).

##### TMR cues

For the paired associates scored as correct on the final pre-sleep test, half of the verbal cues and half of the non-verbal cues were randomly selected and intermixed for replay in SWS. Two additional control cues that were not present at learning (the spoken word “surface” and the sound of a guitar strum) were randomly interspersed within the memory cues. These were played the same number of times that their corresponding verbal and non-verbal cues were replayed, and at the beginning of the TMR rounds (two verbal and two non-verbal) to ensure that participants’ sleep would not be disturbed during auditory stimulation.

##### Overnight sleep

Lights were turned out at ~ 11 pm. White noise was played throughout the night to habituate participants to auditory stimulation (39 dB). TMR began after participants had exhibited at least 2 min of continuous SWS (as determined via online sleep EEG monitoring). Memory cues were played at 5 s intervals and white noise intensity was lowered during the replay of each cue to promote acoustic clarity. Because the number of memory cues varied across participants, null events (i.e. events with no stimulation) were randomly intermixed between the cues so that each round of TMR always lasted 290 s. TMR was repeated throughout the first two cycles of SWS with 1-min intervals placed between each completed round. Cueing was stopped if participants transitioned from SWS to another sleep stage or wakefulness, or showed signs of microarousals, but was restarted if they returned to SWS. At ~ 7 am, participants were woken up, unless they were in SWS or rapid eye movement (REM) sleep, in which case they were allowed to continue sleeping until they woke up or reached sleep stage N1 or N2.

#### Experiment 2

Experiment 2 followed identical procedures as Experiment 1, with the only exception that the verbal cues were presented in a male voice at training and test, and in a female voice during sleep (the mean ± SEM number of training rounds needed to reach criterion was 1.39 ± 0.12 for verbal cue training and 1.74 ± 0.09 for non-verbal cue training *t*(22) = 2.34; *P* = 0.029).

### Stimuli

#### Verbal cues

Thirty-five monosyllabic and disyllabic words (mean ± SD syllable count = 1.54 ± 0.51) were taken from the University of South Florida (USF) word association, rhyme, and word fragment norms (Nelson et al. 1998; Maki et al. 2004) for use as verbal cues. The words were recorded using two separate speakers; one male and one female. The male and female word recordings were of similar duration (mean ± SD ms: male = 769.29 ± 104.95, female = 774.80 ± 99.14, t(34) = 0.49; *P* = 0.63). An additional word (“surface”) was taken from the USF norms for use as a spoken control cue (male duration = 990 ms; female duration = 950 ms). The abstract nature of this control word was intentional so that it remained distinct from the verbal memory cues.

#### Non-verbal cues

Thirty-five environmental sounds were taken from previous studies (Rudoy et al. 2009; Oudiette and Paller 2013) and from freesound.org for use as non-verbal cues. The sounds were similar in length to both the male and female versions of the verbal cues (mean duration ± SD ms = 740.97 ± 156.29, *F*(2,102) = 0.76; *P* = 0.47). Additionally, the sound of a guitar strum (524 ms) was taken from Rudoy et al. (2009) to serve as the non-verbal control cue.

#### Visual stimuli

A further seventy monosyllabic and disyllabic words were taken from the USF norms for use as visual targets in the in the verbal and non-verbal paired associates. Each word was paired with a verbal and non-verbal auditory cue, resulting in two 35-item sets of verbal paired associates (verbal A and B) and two 35-item sets of non-verbal paired associates (non-verbal A and B). For the experiments, if the verbal paired associates were taken from set A, the non-verbal paired associates were taken from set B and vice-versa (counterbalanced across participants). None of the paired associates had a clear semantic link. Out of the sets of 35 verbal/non-verbal paired associates, 3 pairs were allocated to practice trials, 4 were allocated to filler trials, and the remaining 28 pairs of each stimulus type were allocated to the main task.

### Equipment

#### Sleep electroencephalography (EEG)

Sleep was monitored using an Embla N7000 PSG system with RemLogic version 3.4 software. Gold-plated electrodes were attached to the scalp in accordance with the international 10-20 system at frontal (F3 and F4), central (C3 and C4), and occipital (O1 and O2) locations, and were each referenced to the contralateral mastoid (M1 and M2). Left and right electrooculography electrodes were attached, as were electromyography electrodes at the mentalis and submentalis bilaterally, and a ground electrode was attached to the forehead. Each electrode had a connection impedance of < 5 kΩ and all online signals were digitally sampled at 200 Hz. Sleep scoring was conducted on the referenced central electrodes (C3 and C4) in accordance with standardized criteria (Iber 2007).

#### Targeted memory reactivation

TMR was implemented with Presentation v17.0 (Neurobehavioral Systems, Inc.). Auditory cues were played via a speaker placed ~ 1.5 m above the bed, which was connected to an amplifier in a separate control room.

### E‌EG analyses

All EEG data preprocessing and analyses were conducted in MATLAB version 2019a using FieldTrip toolbox version 10/04/18 (Oostenveld et al. 2011) and EEGLAB 2023 (Delorme and Makeig 2004).

#### Preprocessing

Sleep EEG data were re-referenced to the linked mastoids (average of M1 and M2), notch filtered at 49–51 Hz, high-pass filtered at 0.5 Hz, and then segmented into trials (−1 to 3.5 s around cue onset). Using FieldTrip’s Databrowser function, the data were first visually inspected for noisy channels (none were identified). Automatic artifact rejection was then implemented using FieldTrip’s automated artifact rejection function (*ft_artifact_zvalue*). In this step, muscle artifacts at 15–32 Hz (Brunner et al. 1996) were exaggerated using filters and *z*-transformations (0.1-s padding on each side of the artifact) and then removed (mean ± SD trials rejected across all participants in both experiments = 3.96 ± 2.26). The remaining artifacts were manually rejected based on visual inspection via FieldTrip’s Databrowser (mean ± SD noisy trials rejected across all participants in both experiments = 4.14 ± 5.23). Trials that fell outside of sleep stages N2 or SWS were excluded prior to analysis (mean ± SD trials removed across all participants in both experiments = 7.55 ± 10.84). Table 1 shows the number of trials in each condition after artifact rejection and trial removal.

**Table 1:** **TMR trials per condition**.

	**Memory cues**		**Control cues**	
	**Experiment 1**	**Experiment 2**	**Experiment 1**	**Experiment 2**
**Verbal**	*Matched* 89.68 ± 35.60	*Mismatched* 78.22 ± 29.37	*Matched* 108.50 ± 41.81	*Mismatched* 97.17 ± 33.82
**Non-verbal**	88.57 ± 38.85	79.57 ± 32.18	107.64 ± 46.83	97.70 ± 37.45
**Total**	169.02 ± 66.58		206.55 ± 78.93	

Control cues were intermixed with the memory cues and also played at the beginning of each TMR set to ensure that participants’ sleep would not be disturbed during auditory stimulation (hence a higher number of control cues than memory cues). Verbal memory and control cues were presented in the same voice as at learning (matched) in Experiment 1 and in a different voice to learning in Experiment 2 (mismatched). Data are shown as mean ± SD.

#### Time-frequency analyses

Time-frequency representations (TFRs) were calculated for frequencies ranging from 4 to 30 Hz. Data were convolved with a 5-cycle Hanning taper in 0.5-Hz frequency steps and 5-ms time steps using an adaptive window-length (i.e. where window length decreases with increasing frequency, e.g. 1.25 s at 4 Hz, 1 s at 5 Hz, etc.). TFRs were converted into % power change relative to a − 0.3 to −0.100 s pre-cue baseline window. This window was chosen to mitigate baseline contamination by post-stimulus activity while preserving proximity to cue onset (Cairney et al*.* 2018a).

#### Event-related potentials

For event-related potentials (ERPs), data were high-pass filtered at 0.5 Hz and low-pass filtered at 30 Hz. Data were baseline-corrected relative to a −0.2 to 0 s pre-cue window to preserve proximity to cue onset (Cairney et al*.* 2018a).

#### Spindle detection

Spindles were automatically detected at each channel using a wavelet-based detector (Wamsley et al. 2012; Mylonas et al. 2020a; Denis et al. 2021). The continuous raw data were re-referenced to the linked mastoids (average of M1 and M2), notch filtered at 49–51 Hz, high-pass filtered at 0.5 Hz, and subjected to a time-frequency decomposition using complex Morlet wavelets. The peak frequency of the wavelet was set to 13.5 Hz, with a 3-Hz bandwidth (i.e. the 12–15 Hz “fast” spindle range). Spindles were identified by applying a thresholding algorithm to the extracted wavelet scale (*fun_sleep_spindles*). A spindle was detected whenever the wavelet signal exceeded a threshold of nine times the signal median for at least 0.4 s (Denis et al. 2022). This threshold has been empirically determined to maximize between-class (spindle vs non-spindle) variance (Mylonas et al. 2020b). Peri-event histograms were created to examine the timing of spindle events following verbal and non-verbal cues. Each detected spindle was binned into time segments based on its peak amplitude relative to cue onset (t0) in 0.5 s time-bins (T1: 0–0.5 s, T2: 0.5–1 s, T3: 1–1.5 s, T4: 1.5–2 s, T5: 2–2.5 s).

#### Statistics

ERP and TFR analyses were corrected for multiple comparisons using FieldTrip’s non-parametric cluster-based permutation method with 1000 randomizations (increased to 1500 when the standard deviation of the *P*-value crossed the alpha-value; Meyer et al. 2021). All time-frequency clusters were defined by channel^*^time^*^frequency (cluster threshold *P* < 0.05, two-tailed). The time window of interest in the TFR was 0.3–2.5 s (Cairney et al*.* 2018a). ERP clusters were defined by time (averaged across channels) and based on a 0–2.5 s time window of interest (4–30 Hz, cluster threshold *P* < 0.05, two-tailed).

Because Experiments 1 and 2 were highly similar (with the only difference being the match vs mismatch of speaker between training and verbal TMR), we first collapsed the data across experiments and compared memory cues to control cues with a dependent-samples analysis. To assess the effects of cue type (verbal vs non-verbal) on oscillatory activity, we used a factorial approach. We calculated the grand average difference for the contrast [memory cues > control cues] within each condition (verbal cues and non-verbal cues), and then entered these contrasts into a dependent-samples analysis (verbal^memory cues > control cues^ > non-verbal^memory cues > control cues^). We also used a factorial approach to assess the effects of matched vs mismatched verbal cues (i.e. matched^memory cues > control cues^ > mismatched^memory cues > control cues^). However, because the matched and mismatched conditions were collected across Experiments 1 and 2, respectively, we used an independent-samples comparison in this analysis.

Because we did not have a priori hypotheses, Cohen’s *d*_z_ effect sizes were based on the largest identified clusters by averaging power across the time points, frequencies and channels that contributed to the clusters at any point. More specifically, we computationally outlined the cluster with a rectangle defined by the minimum and maximum time and frequency. We selected only the channels which contributed to the cluster and calculated the effect size of the averaged data within these rectangles (Meyer et al. 2021; see Figs S1a and S4).

To investigate peak amplitude timing of spindles evoked by verbal and non-verbal cues, we fitted a linear mixed effects model for each electrode to test for the effects of condition (verbal [memory>control cues]/non-verbal [memory>control cues]) and time bin (T1, T2, T3, T4, T5; see above), their interaction as fixed effects and participant as a random effect (spindles ~ condition × time-bin) + (1 | participant). We used a cluster-based permutation approach across electrodes to address issues with multiple comparisons (similar to the approach used by Mylonas et al*.* 2020a). For each term in the model, clusters were formed from adjacent electrodes that met an uncorrected threshold of *P* < 0.05. Permutation distributions were created by randomly shuffling the labels (Condition, Time) 1000 times at each electrode and retaining the cluster with the maximum statistic for each permutation. Follow-up pairwise *t*-tests were performed as appropriate and performed on data averaged across significant electrodes in the overall cluster.

## Results

### Memory cues and control cues

**Fig. 2:**
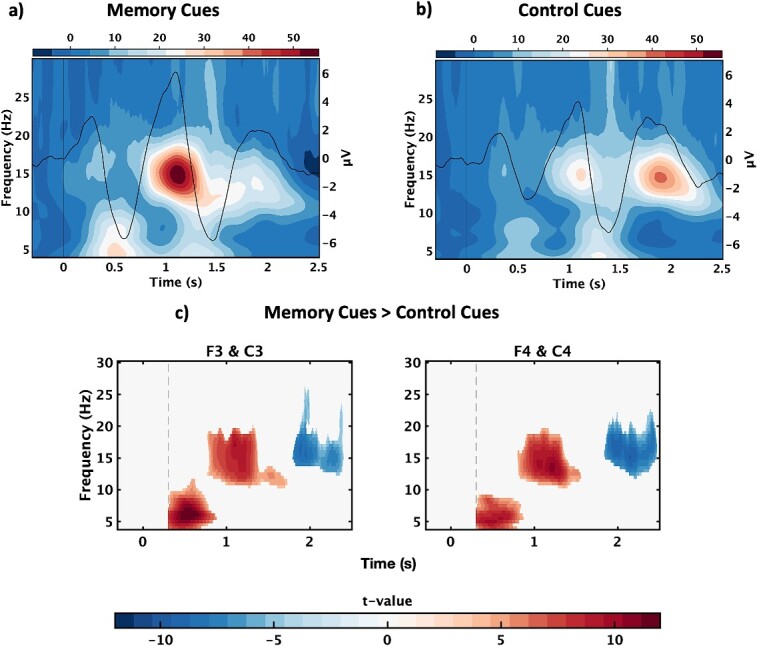
Memory cues and control cues. Grand average time-frequency representations with superimposed event-related potentials (baseline corrected and averaged across all channels) for (a) memory cues and (b) control cues. Color bars represent % change. (c) T-value map for the largest clusters from the memory cue > control cue comparison shown in time-frequency space, separately for the left and right hemispheres. The dashed line represents the onset of the statistical window. Data are collapsed across Experiments 1 (verbal vs non-verbal memory cues) and 2 (acoustically matched vs mismatched verbal memory cues).

First, we examined the sleeping brain’s response to memory cues (Fig. 2a) and control cues (Fig. 2b). Significant differences in the time-frequency representation (TFR) were observed for memory cues (vs control cues, *P* < 0.05, Fig. 2c). The identified clusters showed an increase in theta/alpha power (~4–11.5 Hz) across both hemispheres at ~ 0.3–0.9 s (F3 & C3: *d*_z_ = 0.56, F4 and C4: d_z_ = 0.48), which was followed by an increase in spindle/beta power (~10.5–20 Hz) at ~ 0.8–1.7 s (F3 and C3: *d*_z_ = 0.51, F4 and C4: *d*_z_ = 0.53). There was also a later decrease in power across a wider spindle/beta band (~12–26 Hz) in both hemispheres (~1.8–2.5 s, F3 and C3: *d*_z_ = −.39, F4 and C4: *d*_z_ = −.46, Fig. 2c and 2d), though this corresponded to an increase in spindle power for control cues. Because the coupling between spindles and slow oscillations has been implicated in memory consolidation, we examined the phase-amplitude coupling between slow-oscillations and spindles. However, there were no significant differences between the memory cue and control cue conditions (see Supplementary Materials for methods, Fig. S1b).

It is important to note that the memory cue ERP (Fig. 2a) was significantly stronger than the control cue ERP (Fig. 2b, p < .05), with three clusters at ~ 0.4–0.7 (negative, d_z_ = −.70), ~ 0.9–1.3 (positive, d_z_ = 0.59), and ~ 1.4–1.8 s (negative, d_z_ = −.64, Fig. S3a). Any difference in the time-frequency response between memory and control cues could thus be attributed to habituation (because the control cues were always the same, whereas the memory cues encompassed a wide range of verbal and non-verbal stimuli). However, separate analyses of the time-frequency representation (memory cues > control cues) in the first and second half of the TMR window (where habituation effects would presumably be stronger in the second half) revealed highly similar patterns of results (Fig. S2), suggesting that our time-frequency results cannot be attributed to habituation alone.

### Verbal and non-verbal memory cues

**Fig. 3:**
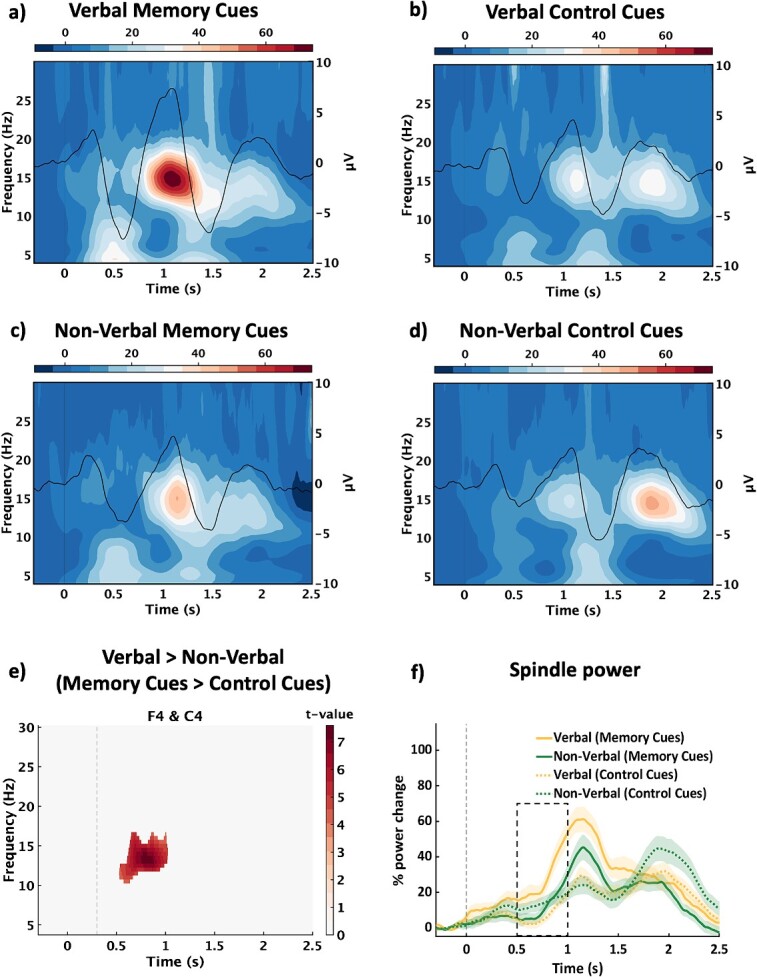
Verbal cues and non-verbal cues. Grand average time-frequency representations with superimposed event-related potentials (baseline-corrected and averaged across all channels) for (a) verbal memory cues, (b) verbal control cues, (c) non-verbal memory cues, and (d) non-verbal control cues. Color bars represent % change. (e) T-value map for the largest clusters from the verbal > non-verbal (memory cue > control cue) comparison shown in time-frequency space. The dashed line represents the onset of the statistical window. (f) Mean (±SEM) power change over time for all four conditions (collapsed across the channels [F4 and C4] and frequencies [10.5–16.5 Hz] that contributed to the largest cluster in (e). The rectangle illustrates the approximate timing that contributed to the cluster. Data are collapsed across Experiments 1 (verbal vs non-verbal memory cues) and 2 (acoustically matched vs mismatched verbal memory cues).

Next, we examined whether verbal and non-verbal memory cues evoke distinct patterns of oscillatory activity during sleep. We subtracted the evoked control cue response (Fig. 3b and 3d) from the memory cue response (Fig. 3a and c), separately for verbal and non-verbal cues, leading to a 2x2 factorial design (verbal^memory cues > control cues^ > non-verbal^memory cues > control cues^). A significant difference emerged (*P* < 0.05), corresponding to an increase in spindle activity (~10.5–16.5 Hz) across the right hemisphere (F4 & C4) at ~ 0.5–1 s (d_z_ = 0.27, Fig. 3e and f). Interestingly, post-hoc tests (which included data from the time and frequency limits and the channels contributing to the cluster, see Fig. S4) revealed a stronger spindle response for verbal memory cues relative to both non-verbal memory cues (*P* = 0.008) and verbal control cues (*P* < 0.001). No significant differences were observed between the non-verbal memory cues and non-verbal control cues (*P* = 0.800), nor between the verbal and non-verbal control cues (*P* = 0.129, all Bonferroni corrected). Note that the same results also emerged when restricting our analysis to participants who completed the same number of training rounds for verbal and non-verbal paired associated prior to sleep (Fig. S5).

To examine the temporal characteristics of spindle events arising from verbal and non-verbal memory cues (vs control cues), spindle peak amplitudes were grouped into 5 time-bins (0.5, 0.5–1, 1–1.5, 1.5–2, and 2–2.5 s after cue onset). We then fitted a linear mixed effects model (spindles ~ condition × timebin) + (1 | participant) to test for the effects of condition (verbal^memory cues > control cues^ > non-verbal^memory cues > control cues^) and time bin on spindle peak amplitude. This revealed a significant interaction, with an identified cluster including all six channels (*P* < 0.05). Post-hoc pairwise comparisons revealed that spindles peaked more frequently for verbal cues than non-verbal cues in an early time-bin (0.5–1 s; *P* < 0.001, *d* = 1.06; Table S1).

To examine whether there was any difference in ERPs for the verbal and non-verbal cues, we applied to our ERP analysis the same factorial approach as that used in our time-frequency analysis (verbal^memory cues > control cues^ > non-verbal^memory cues > control cues^). We observed a significant difference (*P* < 0.05), with two clusters at ~ 0.8–1 (positive, d_z_ = 0.47) and ~ 1.2–1.5 s (negative, d_z_ = −.50, Fig. S3b).

### Matched and mismatched memory cues

Finally, we investigated whether verbal memory cues that are acoustically matched to those heard at learning evoke distinct neural responses to those arising from mismatched verbal cues (i.e. same vs different voice). We subtracted the evoked control cue response from the memory cue response, separately for the matched (Experiment 1) and the mismatched conditions (Experiment 2), leading to a 2 × 2 mixed factorial design (matched^memory cues > control cues^ > mismatched^memory cues > control cues^). However, no significant interaction emerged (Fig. S6).

The same factorial approach was applied to our ERP analysis, but again no significant interaction emerged (Fig. S3c).

## Discussion

Overnight memory consolidation is achieved through the reactivation of newly formed memory traces in SWS (Born and Wilhelm 2012; Rasch and Born 2013; Klinzing et al. 2019; Staresina 2024). Studies employing the TMR paradigm have provided crucial support for this hypothesis, as well as the view that sleeping brain rhythms, including theta and spindle oscillations, play a central role in overnight memory processing (Schreiner and Rasch 2017; Antony et al. 2019). In the current study, we explored whether neural activity evoked by TMR in SWS is influenced by (i) the acoustic properties of memory cues and (ii) the acoustic overlap between cues presented at learning and sleep.

Relative to previously unheard control cues, memory cues prompted an increase in theta/alpha and then spindle power. Importantly, the evoked spindle response was significantly stronger for verbal memory cues than non-verbal memory cues, suggesting that verbal auditory stimuli might be particularly potent triggers of memory reactivation in sleep. There were no significant differences in the evoked response to verbal cues that were acoustically matched or mismatched to learning.

The observed increase in theta/alpha and then spindle power for memory cues relative to control cues is in keeping with a number of prior studies that examined the neural correlates of TMR (Schreiner et al*.* 2015a; Lehmann et al. 2016; Groch et al. 2017; Cairney et al*.* 2018a; Laventure et al. 2018; Göldi et al. 2019; Bar et al. 2020; Schechtman et al. 2021). Based on these findings, it has been argued that theta and spindle rhythms play complementary roles in overnight consolidation, with the former supporting memory reinstatement and the latter facilitating memory strengthening and stabilization (Schreiner and Rasch 2017). These previous studies reporting an increase in delta/theta and spindle activity during TMR have used a wide variety of memory cues, including auditory (Schreiner et al. 2015a; Groch et al. 2017; Cairney et al. 2018a; Göldi et al. 2019; Schechtman et al. 2021) and olfactory stimuli (Laventure et al. 2018; Bar et al. 2020), and have assessed the retention of memories with varying emotional properties (Lehmann et al. 2016) across both declarative (Schreiner et al. 2015a; Lehmann et al. 2016; Groch et al. 2017; Cairney et al. 2018a; Göldi et al. 2019; Bar et al. 2020; Schechtman et al. 2021) and non-declarative domains (Laventure et al. 2018). Hence, spindle and theta oscillations appear to play a central role in sleep-associated memory reactivation and consolidation.

The increase in spindle activity observed during TMR was amplified for verbal relative to non-verbal memory cues. Importantly, post-hoc tests revealed a significant increase in spindle activity for verbal memory cues relative to verbal control cues (and non-verbal memory cues), but no such difference between verbal and non-verbal control cues, suggesting that our findings do not simply reflect generalized differences in the processing of environmental sounds and spoken words. Along the same lines, when analyzing the temporal characteristics of evoked spindle responses, we found that spindle amplitudes peaked more often in an early time window for verbal relative to non-verbal memory cues. Given the putative function of spindles in sleep-associated memory processing (Schreiner and Rasch 2017; Antony et al. 2019), these findings suggest that both verbal and non-verbal memory cues are effective triggers of memory reactivation in sleep, but verbal memory cues might ignite these processes more rapidly, given the early spindle response.

Why might verbal cues be more effective at reactivating memories than non-verbal cues? Based on current understanding of the phonological and semantic processes underpinning spoken word recognition during wakefulness (Gaskell and Mirkovic 2016; McMurray et al. 2022), it is possible that the sleeping brain has better access to meaning when presented with verbal relative to non-verbal memory cues, with an enhanced spindle response reflecting engagement of multi-level decoding pathways during retrieval. Indeed, previous work has suggested that the brain can process semantic information in sleep (Bastuji et al. 2002; Wislowska et al. 2022) and even learn new verbal associations (Züst et al. 2019; Schmidig et al. 2022; Xia et al. 2023). This increased access to meaning might in turn facilitate the reactivation and stabilization of relevant memory traces. Another, not mutually exclusive, possibility is that the verbal stimuli (i.e. spoken words) in the present study were more similar to the targets (i.e. written words) than the non-verbal stimuli and might therefore more easily reinstate the associated memory. Thus, it may not be that verbal stimuli on their own are more effective at reactivating memories than non-verbal stimuli, but that the similarity between the cue and target are the important factor.

We observed no significant differences in the evoked EEG response for verbal memory cues that were acoustically matched or mismatched to those presented at learning (based on the speaker’s voice). This is at odds with our behavioral data (Cairney et al. 2017), which show the typical selective benefit of TMR for cued memories in the matched condition, but a generalized benefit for all cued and non-cued memories in the mismatched condition. Recent work has shown that, although TMR delivered in SWS evokes an increase in delta/theta (0.5–8 Hz) and spindle (11–16 Hz) power irrespective of whether the memory cues are associated with one or multiple targets, the magnitude of this increase is modulated by the number of targets (Schechtman et al. 2021). Hence, differences in the evoked response to matched and mismatched cues might be observed as gradual increases in power (according to the number of potential targets reactivated by a mismatched cue). Because our matched and mismatched conditions comprised different groups (from Experiments 1 and 2, respectively), we were unable to address this possibility in the current dataset, but it remains an open question for future research.

It should be noted that Cairney et al. (2017) did not find significant differences in paired associates forgetting between the verbal and non-verbal cueing conditions, nor any relationship between the overall TMR cueing benefit and spindle density. However, the measure of paired associates forgetting used in Cairney et al. was fairly coarse in comparison to measures used in prior studies implicating spindle activity in the memory benefits of TMR (e.g. visuospatial retrieval; Cairney et al. 2014; Creery et al. 2015). Future work can thus assess the relative impacts of verbal and non-verbal memory cues in the context of finer-grained behavioral measures.

It is also important to note that our paradigm included only two control cues (one verbal and one non-verbal), which were repeatedly replayed and intermixed with a much larger number of distinct memory cues. Event-related potentials were thus significantly smaller for control cues than memory cues, reflecting habituation of the neural response that might have influenced our other findings (Megela and Teyler 1979). However, control analyses separating earlier and later cueing trials showed a comparable increase in spindle activity for memory cues relative to control cues, suggesting that habituation is unlikely to account for our time-frequency results. Future research comparing neural signatures of memory cues and control cues should ensure that the control cues are optimally designed, by matching the two types of stimuli in terms of auditory and linguistic characteristics, as well as their frequency of repetition. Finally, given the exploratory nature of this secondary analysis, further studies are needed to confirm our findings with an a priori hypothesis-driven approach.

## Conclusion

In conclusion, we found that memory cues evoke increases in theta and spindle power, which have been previously linked to memory reinstatement and stabilization during sleep. We also showed, for the first time, that the TMR-evoked spindle response is stronger for verbal memory cues than non-verbal memory cues, suggesting that verbal stimuli are more effective triggers of memory reactivation in the sleeping brain. However, we did not observe any differences when comparing neural responses to verbal memory cues that were matched or mismatched to those encountered at learning. Taken together, these findings provide novel insights into how the sleeping brain processes memory cues with distinct acoustic properties.

## Supplementary Material

Guttesen_Supplementary_Materials_R1_bhae183

## Data Availability

The data used to produce the results and figures along with associated scripts has been made available on the Open Science Framework: https://osf.io/6zpdg/
