# Supplementary material for: Delineating memory reactivation in sleep with verbal and non-verbal retrieval cues
Source: Cereb Cortex. 2024 May 14;34(5):bhae183. doi: 10.1093/cercor/bhae183 (PMC11094403; doi:10.1093/cercor/bhae183)
Supplement: Guttesen_Supplementary_Materials_R1_bhae183 [file guttesen_supplementary_materials_r1_bhae183.docx]

# **Supplementary materials**

Delineating memory reactivation in sleep with verbal and non-verbal retrieval cues

*Slow oscillation/spindle coupling*

Slow oscillations (SOs) were detected at every electrode using an automated algorithm (*fun_slow_oscillations*) and CircStatToolbox (version 1.21.00, 2012a; Berens 2009; Denis et al. 2021). First, data were bandpass filtered between 0.5-4 Hz and all positive-to-negative zeros crossing were identified. SOs were detected whenever two such consecutive zero crossing fell 0.8-2 seconds apart, corresponding to 0.5-1.25 Hz. To identify coupling events using *fun_so_spindle_coupling* (Denis *et al.* 2021), EEG data were bandpass filtered between 12-15 Hz. Then, the Hilbert transform was applied to extract the instantaneous phase of the delta (0.5-4 Hz) filtered signal and the instantaneous amplitude of the sigma (12-15 Hz) filtered signal. For each detected spindle, the peak amplitude of that spindle was determined. It was then determined whether the spindle peak occurred within the time course (i.e., between two positive-to-negative zero crossings) of any detected SO. If the spindle peak was found to occur during a SO, the phase angle of the SO at the peak of the spindle was determined. We extracted the average coupling phase (in degrees) and coupling consistency, measured as the mean vector length. Further analysis of spindle and SO data only included trials which were also included in the TFR analyses (see Table 1 in the main manuscript).

To compare condition differences in SO-spindle coupling, coupling metrics were first averaged over frontal and central electrodes. Due to not having any coupling events in one condition, three participants were excluded from further analysis (resulting in N=48). Differences in coupling phase were assessed using Hotelling’s paired samples test for circular data. Differences in coupling consistency were assessed using a paired samples t-test.


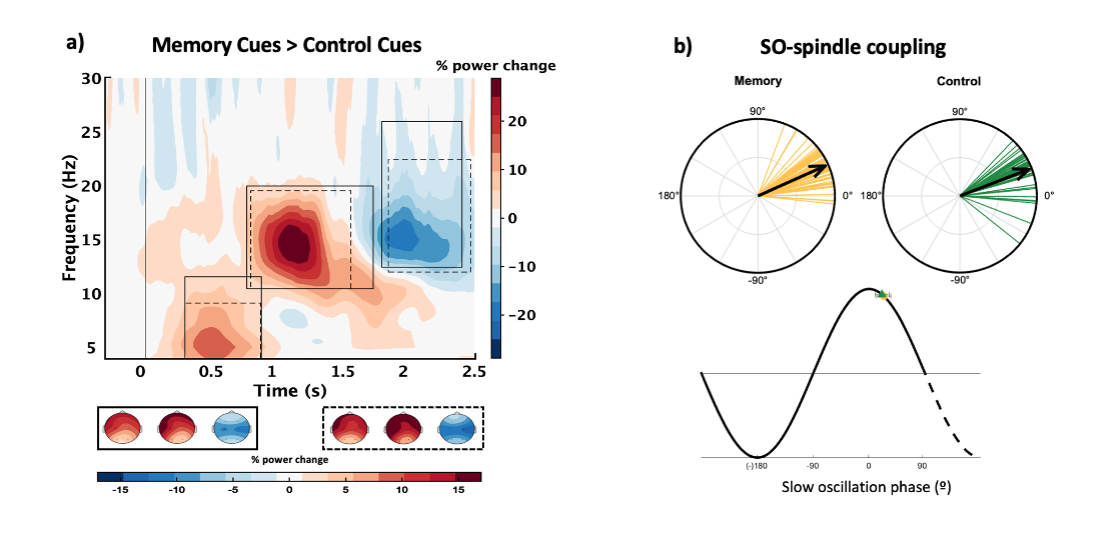
**Figure S1.** **Memory Cues > Control Cues.** **a)** Time-frequency and topographical plot representing power differences [Memory Cues > Control Cues]. The rectangular shapes circumscribe the time and frequency limits of the clusters which were used for the effect size calculations (separated into left and right hemisphere, solid and dashed lines, respectively). The rationale for this circumscribed rectangle approach is that the precise shape of any cluster is not very robust across analyses, and effect sizes based on the precise shape of the cluster may be of limited use for future research (Meyer et al. 2021). **b)** Phase-amplitude coupling between SOs (0.5-4 Hz) and spindles (12-15 Hz) for the memory cues and control cues, where 0 represents the peak of the SOs (averaged across F3, F4, C3, C4). No significant differences between cue type were observed.


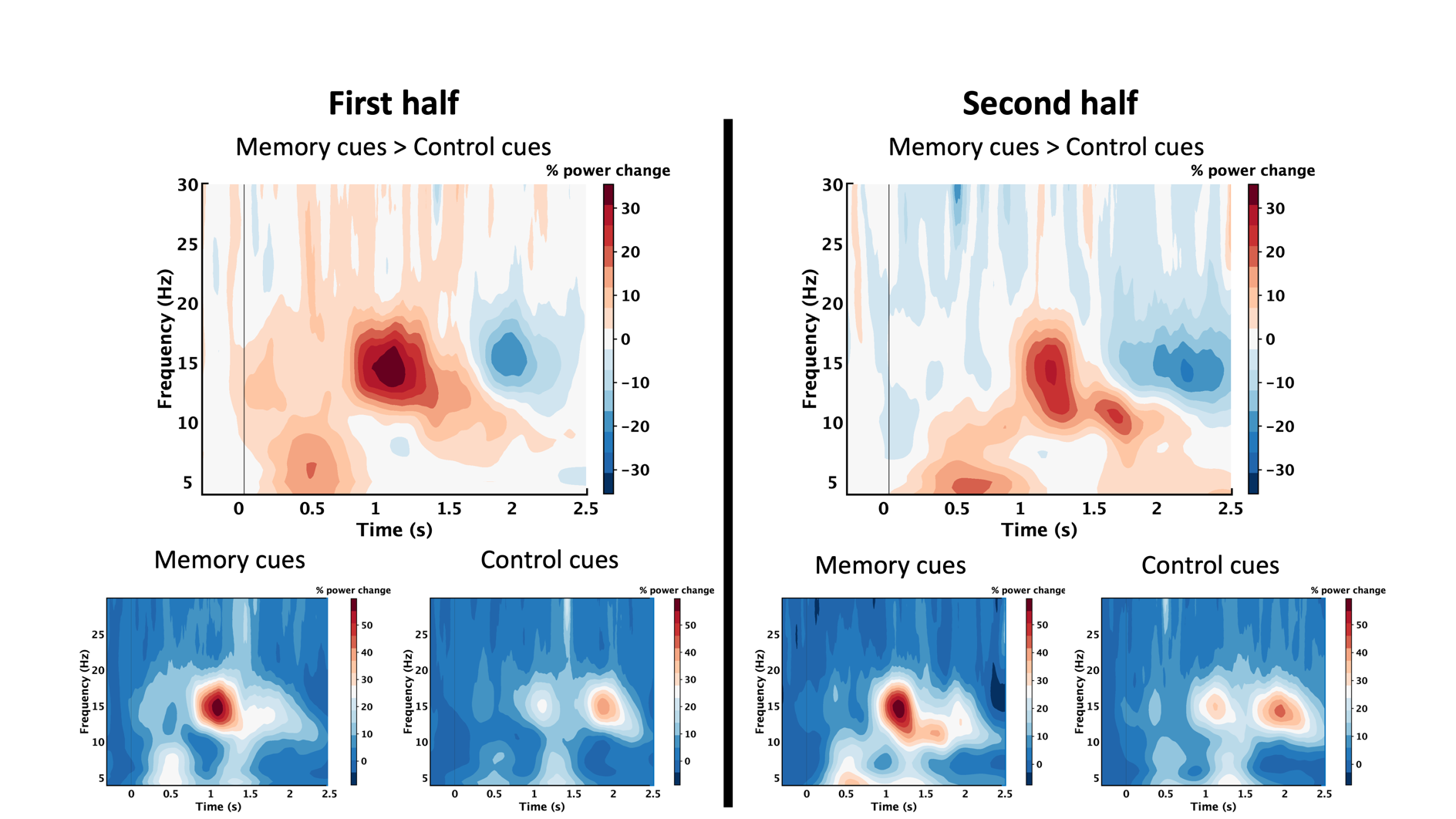


**Figure S2**. Memory cues and control cues in the first (left) and second (right) half of the TMR trials. Grand average time-frequency representations baseline corrected and averaged across all channels) for memory cues > control cues (top) and plotted separately for memory cues and control cues (bottom) showing power changes.


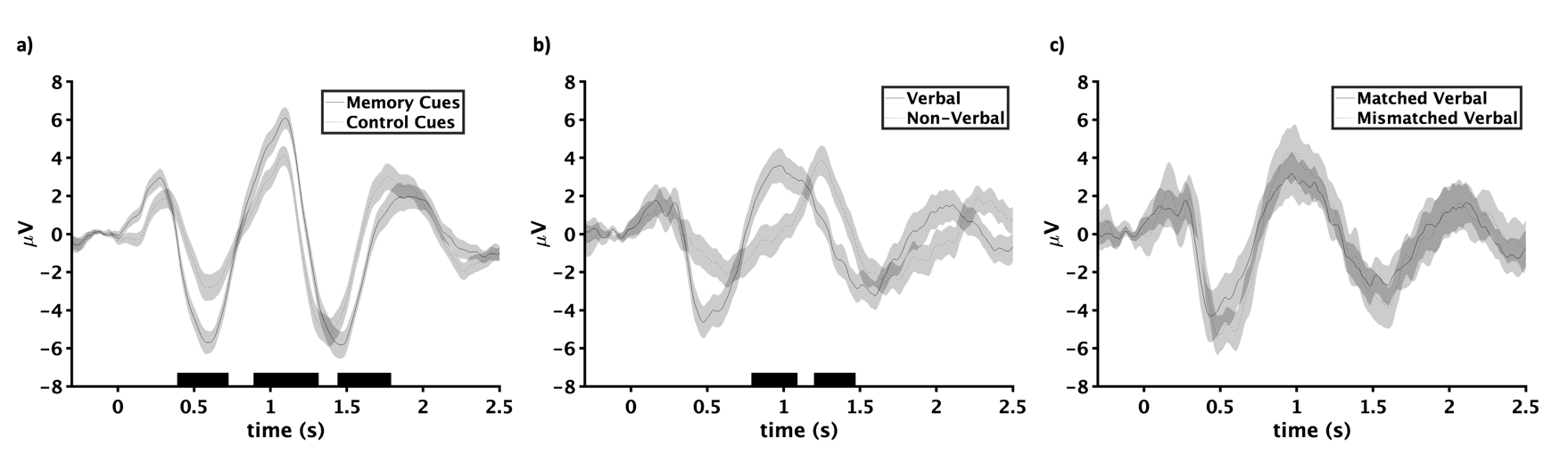


**Figure S3**. Grand average (mean ±SEM) event-related potentials (baseline corrected and averaged across all channels) for **a)** all memory cues and control cues, **b)** verbal cues and non-verbal cues (memory cues > control cues), and **c)** matched and mismatched speaker (memory cues > control cues). The black boxes at the bottom reflect timings of the identified clusters.


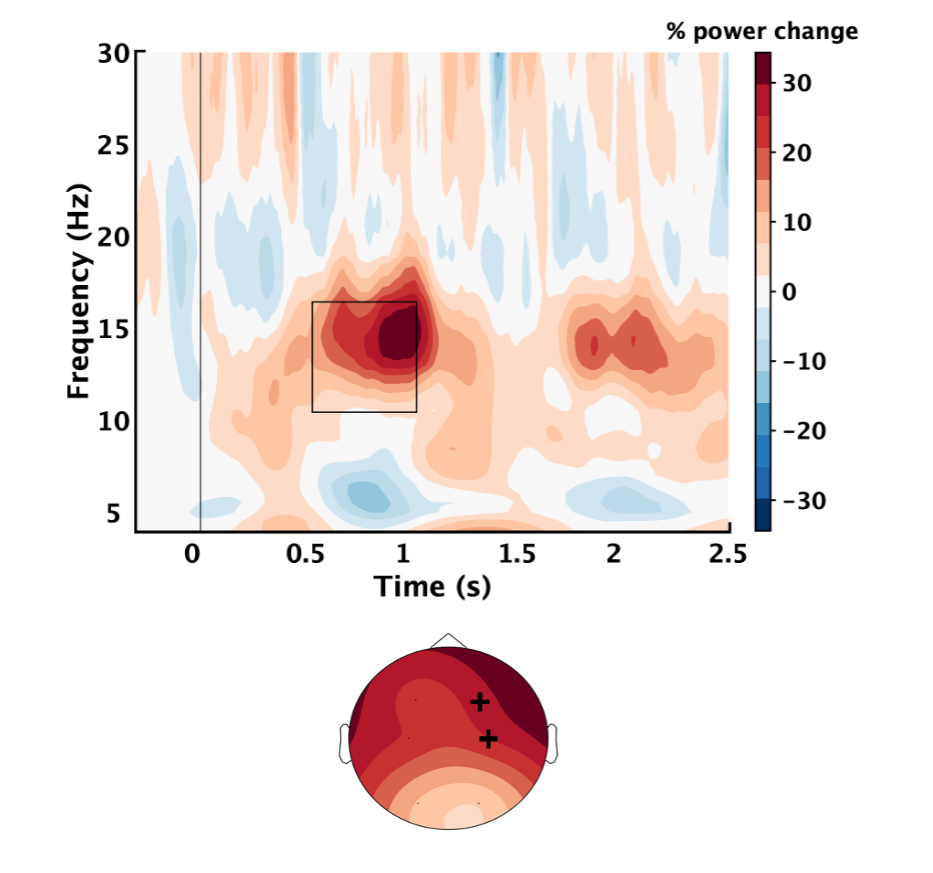


**Figure S4**. **Verbal Cues > Non-Verbal Cues (Memory Cues > Control Cues).** Time-frequency and topographical plot representing power differences (verbal cues > non-verbal cues [memory > control cues]). The rectangular shape circumscribes the time and frequency limits of the cluster which was used for the post-hoc analyses and effect size calculations.


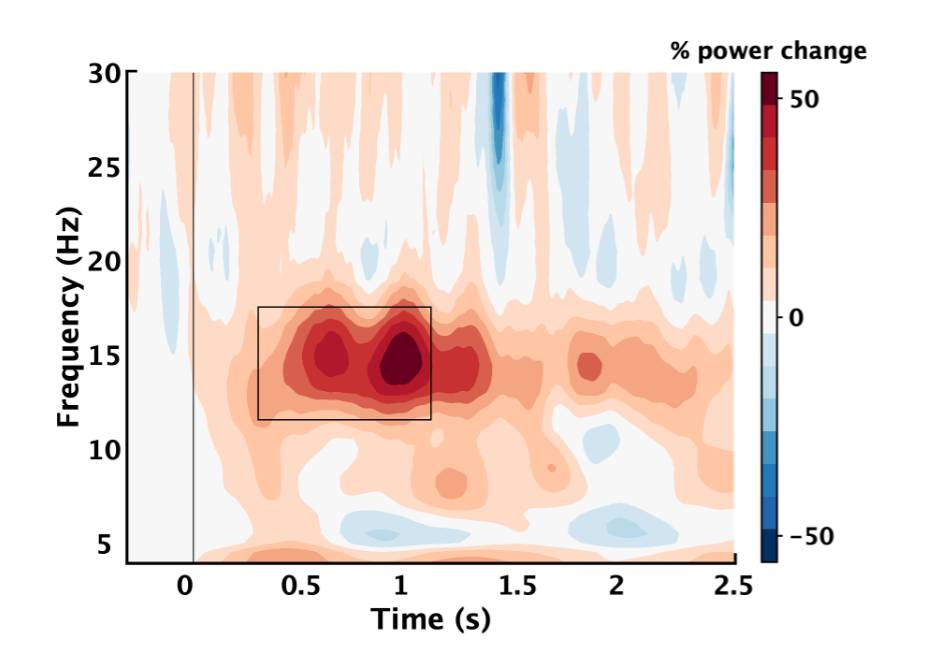


**Figure S5**. Time-frequency plot showing verbal cues > non-verbal cues (memory cues > control cues) for subsample of participants (N=24) who had the same number of training rounds for verbal and non-verbal paired associates prior to sleep. The cluster-based permutation analysis revealed a similar result to our main analysis, showing a significant difference between the conditions (p < .05), with a corresponding increase in spindle activity for verbal memory cues (~11.5-17.5 Hz) across the right hemisphere (F4 & C4) at ~0.3-1.1 s (d_z_ = .56).

**Table S1.** Distribution of spindle peak times following verbal and non-verbal memory cues and control cues. Data are shown in mean (± SD) percentage terms across each 0.5 s time-bin.

|  | **0-0.5 s** | | | **0.5-1 s** | | **1-1.5 s** | | **1.5-2 s** | | | **2-2.5 s** | |
| --- | --- | --- | --- | --- | --- | --- | --- | --- | --- | --- | --- | --- |
|  | *Memory* | *Control* | *Memory* | | *Control* | *Memory* | *Control* | *Memory* | *Control* | *Memory* | | *Control* |
| **Verbal  Cues** | 23.77 (14.73) | 18.81  (13.37) | 42.30  (19.88) | | 18.66  (10.56) | 23.21  (14.11) | 28.50  (16.43) | 10.70  (15.35) | 20.44  (16.20) | 0  (0) | | 13.59  (11.41) |
| **Non-verbal Cues** | 21.48  (12.22) | 16.74  (8.97) | 18.16  (9.67) | | 23.45  (13.18) | 23.59  (14.24) | 21.15  11.76) | 25.33  (12.56) | 26.60  14.96) | 11.45  (6.79) | | 12.06  (11.41) |

Note. Values express % of all spindles whose peak amplitude fell within each time bin.


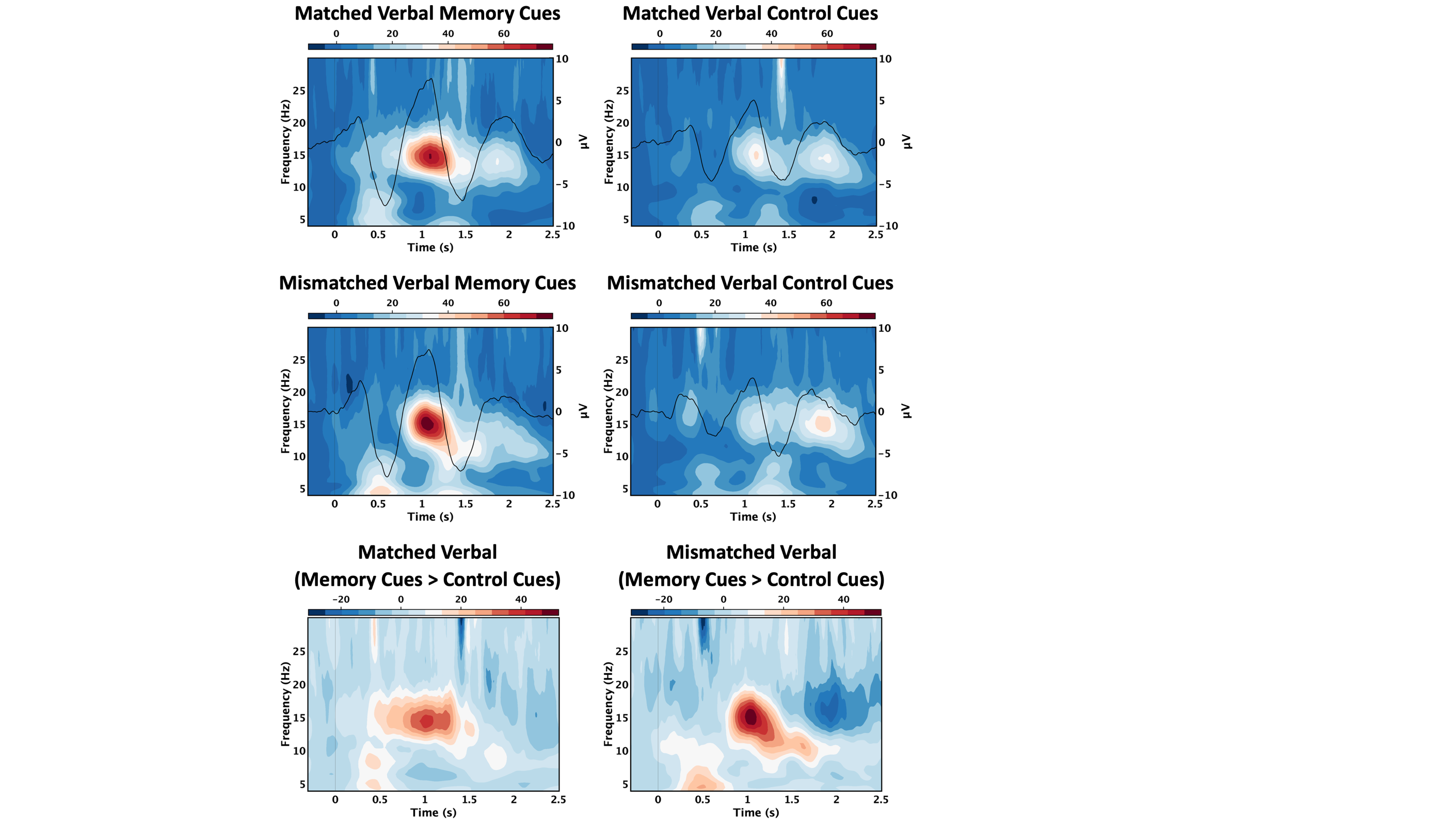


Figure S6. Matched and mismatched verbal cues. Grand average time-frequency representations for the matched verbal cues (Experiment 1) and mismatched verbal cues (Experiment 2) plotted separately for memory cues and control cues with superimposed ERPs (top) and memory cues > control cues (bottom, baseline corrected and averaged across all channels). No significant effects were observed (p > .05). Colour bars represent % change.

**References**

Berens P. 2009. CircStat: a MATLAB toolbox for circular statistics. J Stat Softw. 31:1-21.

Denis D, Mylonas D, Poskanzer C, Bursal V, Payne JD, Stickgold R. 2021. Sleep spindles preferentially consolidate weakly encoded memories. J Neurosci. 41:4088-4099.

Meyer M, Lamers D, Kayhan E, Hunnius S, Oostenveld R. 2021. Enhancing reproducibility in developmental EEG research: BIDS, cluster-based permutation tests, and effect sizes. Dev Cogn Neurosci. 52:101036.
